# Physician factors associated with medical errors in Norwegian primary care emergency services

**DOI:** 10.1080/02813432.2021.1973240

**Published:** 2021-10-07

**Authors:** Svein Zander Bratland, Valborg Baste, Knut Steen, Esperanza Diaz, Gunnar Tschudi Bondevik

**Affiliations:** a National Centre for Emergency Primary Health Care, NORCE Norwegian Research Centre, Bergen, Norway; b Department of Global Public Health and Primary Care, University of Bergen, Bergen, Norway; c Norway & Unit for Migration and Health, Norwegian Institute of Public Health Oslo, Bergen, Norway; d Department of Global Public Health and Primary Care, University of Bergen & National Centre for Emergency Primary Health Care, NORCE Norwegian Research Centre, Bergen, Norway

**Keywords:** Emergency medical services, general practice, general practitioners, health services research, medical audit, medical errors, patient complaints

## Abstract

**Objective:**

The aim of this study was to examine the associations between characteristics of physicians working in primary care emergency units (PCEUs) and the outcome of assessments of the medical records.

**Design:**

Data from a previous case-control study was used to evaluate factors related to medical errors.

**Setting:**

Ten Norwegian PCEUs were included.

**Subjects:**

Physicians that had evoked a patient complaint, and a random sample of three physicians from the same PCEU and time period as the physician who had evoked a complaint. Recorded physician characteristics were: gender, seniority, citizenship at, and years after authorization as a physician, specialty in general practice, and workload at the PCEU. Main outcome measures: Assessments of the medical records: errors that may have led to harm, no medical error, or inconclusive.

**Results:**

In the complaint group 77 physicians were included, and in the random sample group 217. In the first group, 53.2% of the medical records were assessed as revealing medical errors. In the random sample group, this percentage was 3.2. In the complaint group the percentages for no-error and inconclusive for the female physicians were 30.8 and 15.4; and for the male physicians 9.8 and 27.3, *p* = 0.027.

**Conclusion:**

In the group of complaints there was a higher percentage with no assessed medical error, and a lower percentage with inconclusive assessments of medical errors, among female physicians compared to their male colleagues. We found no other physician factors that were associated with assessed medical errors. Future research should focus on the underlying elements of these findings.

**Key points**
- Medical errors are among the leading causes of death and they are essentially avoidable. Primary care emergency units are a vulnerable arena for committing medical errors.
- By assessing the medical records of a group of physicians who had evoked a complaint, no differences related to physician factors were revealed in the incidence of medical errors.
- In the group of female physicians, the proportion of no-errors, was higher, and the percentage of inconclusive medical records was lower than for their male colleagues.
- The Norwegian regulations on independent participation in PCEUs may have modulated these results.

## Introduction

Patient safety incidents (PSIs) have been defined as any unintended or unexpected incident(s) that could have, or were judged to have, led to patient harm [1]. Medical errors are the predominant factor in these incidents. These errors may be defined as an act of omission or commission in planning or execution that contributes or could contribute to an unintended result [2]. There is considerable research on medical errors and patient safety in hospital settings. In a meta-analysis, the impact of the different medical specialties could not be explored [3]. On this background, we consider that more knowledge on patient safety in the primary care setting is needed. Our project is aimed at elucidating this through a study material based on patient complaints and a randomized control group of corresponding physicians from the same units and time period.

The occurrence of medical errors in primary care is relatively common [4]. These errors have been considered preventable in more than 90% of detected cases [1]. Out-of-hours consultations are known to be a setting of high risk for patient safety incidents [5]. In primary care, the physicians may face different and varied working conditions. This includes units with several co-workers and solo practices. Diagnostic errors are reported as most common in primary care solo practice due to workload and inability to easily cross-reference with colleagues [6]. This work situation is the regularity in primary care emergency units (PCEUs) in Norway.

Considering the potential of health deterioration following medical errors in an emergency situation, learning from these errors is crucial. User surveys, reporting systems for healthcare, and patient complaints have been utilized [6–8]. Reviewing the medical record (clinical auditing) is mandatory in identifying poor clinical performance [7,9–11]. In its essence, the medical record related to an emergency situation may be deficient in describing the complete course of events. Studying unintentional incidents is consequently demanding [6,7,11]. In the PCEUs quick decisions and immediate actions towards unknown patients without counselling are often required [6,8]. This has induced the hypotheses that communication skills and experience are important factors in minimizing medical errors in these situations. It has been presumed that the perception of being understood may differ related to the physician’s gender, experience, and native language [12–14].

In 2006 a Norwegian study of medicolegal assessments of complaints against general practitioners indicated an association between medical errors and male physicians and physicians with non-Norwegian citizenship [15]. We have chosen to study physician factors that may lead to medical errors in PCEUs. In the first part of the study, we used a case-control design to focus on patient complaints, regardless of medical errors or not [16]. We found that having a general practice, general practitioner (GP) specialty, or a high workload at the PCEU, was associated with a significantly reduced risk of evoking a complaint. Gender, seniority, and not having Norwegian citizenship at the time of authorization as a physician were not significantly associated with the risk of evoking a complaint.

A complaint may be justified or not, and an error may be followed by a complaint or not. To uncover medical errors that may have led to patient harm, we have studied a group of physicians who had elicited a complaint working in PCEUs, and a random sample of physicians from the same PCEUs in the same period. The aim of this part of the study was to examine the associations between characteristics of the physicians working in PCEUs, their workload, and the outcome of the assessment of the medical records in the complaint-group and the random sample group.

## Material and methods

### Study setting

In 2015 Norway had nearly 5.2 million inhabitants. The population density is low. In 2018 the number of PCEUs was 177: 75 covering one municipality and 102 covering more than one. Structural and organizational arrangements are underlying factors in studying PSIs. For general practitioners in Norway participation in out-of-hours service is an additional duty to their regular medical tasks [17]. The qualification requirements for independent participating in this kind of duty, consist of at least 30 months of clinical work after authorization and having had at least 40 work shifts at medical emergency services provided by PCEUs [18].

### Participants and procedure

At the time of planning the study, the consultation rate at the PCEUs in Norway was 260 per 1000 inhabitants per year. A review of 11 studies with different definitions of incidents and data collection methods calculated a rate of 5 to 80 incidents per 100 000 consultations, in which patients were harmed or may have been harmed [19]. Based on these results and the requirements to participate in our study, we decided to include PCEUs that in total covered one-third of the Norwegian inhabitants, living in urban or rural parts of the country. The chosen units covered ∼1.7 million people. To reach this target population, we invited ten PCEUs to participate in this project. Six of these units were serving major cities and four were serving mainly rural areas. We stipulated that from this selection a total of about 250 patient complaints could be received in one year. This corresponds with a retrospective Irish study from out-of-hours GP [3].

PCEUs in Norway use different electronic patient record systems without a common communicating platform. Because of this, a customized computerized data extraction programme for encrypted transmission of data from the medical records had to be developed. This customized computer programme randomly selected three control physicians (i.e. random sample group) for each case-physician (i.e. having evoked a complaint). In this way, records from four different physicians and four consultations were extracted for assessments.

We mainly selected the largest PCEUs with staffing that was expected to be able to handle the number of complaints together with operating the customized computerized data-extraction programme. We assumed that requesting PCEUs at random for participation in the study could have elicited negative answers from a considerable number of PCEUs, that would have to refrain from participation because of lack of personnel to meet the requirements for handling sensitive data. Additionally, difficulties in recruiting PCEUs to participate in registrations studies are caused by understaffed administrations.

The requirements to meet the ethical considerations on the use of sensitive personal data given by the ethics committee, led us to determine that only the larger cities could have the necessary full-time administrative position sizes. One of the city PCEUs had to refuse to participate due to the installation of a new system for electronic record keeping. In choosing counties with rural inter-municipal PCEUs, comparability in terms of population structure and staffing was governing. These PCEUs were granted technical support, if necessary, in handling the extraction programme.

To facilitate a unified approach to this project, each PCEU was visited twice, and given oral and written guidelines for inclusion and exclusion of cases. They were also shown the use of the customized computerized data extraction programme. By this data extraction, we acquired the unique physician identification number (UPIN), and the parameters on workload during the fourteen days before the index consultation.

This process was followed by assessments of the medical records for uncovering any medical error. A complaint was defined as any written utterance of discontent with the physician’s medical measures, sent directly to the PCEU or *via* external authorities. Excluded were complaints solely about rudeness, impoliteness, or poor communication, where it could not be presumed any significant harm to the patient’s health.

The controls were three randomly selected physicians who had been on duty in the fourteen-day period prior to the case consultation. The computer programme selected these three control physicians from the same PCEU as the case physician. Consequently, a case physician could turn up as a control for another case and vice versa.

The medical records were used for information about the physician characteristics and workload. For this, the UPIN was extracted together with the history of work shifts with numbers of patients during the fourteen-day period prior to the index consultation. The information extracted from the medical records was sent encrypted from the project employee to the proprietor of the LSR (Legestillingsregisteret - Norwegian physician position register). From this register information about the physicians was extracted. The LSR does not provide any information on citizenship change. Seniority was defined as the number of years after authorization.

There were challenges in the data collection process, resulting in one-third of the anticipated number of 250 complaints [16]. The data collection started September 1^st^, 2015, and was extended to March 1^st^, 2017.

For all cases and randomized controls, the specified data were accessible in the medical records. The medical history was absent in one record, making the total number in the complaint group 77. The work shift roster at some of the minor units did not have three different physicians to choose from as controls for the fourteen-day period of inclusion, so the total number of controls was 231. The extractions from the LSR reduced the complete data-sets mainly due to unidentifiable UPIN, making 217 medical records in the random sample group available for reviewing. Missing data were only detected in this group (6.1%).

### Assessments

The medical records in both groups were assessed by the first author (SZB), who has 40 years in general practice with 20 years of experience assessing medicolegal cases. A graded normative tool was used consisting of 13 medicolegal cases, ranging from considered potentially harmful to patients to being considered not harmful. This tool is described in a joint report from the Norwegian Board of Health Supervision and the Norwegian Medical Association. Fact boxes are used in presenting the decisive medical factors [20]. In our study, the assessments were based on the different elements of the medical records including the measures implemented by the physicians. The assessments were divided according to this normative tool into three categories:

1. Medical errors that may have led to harm or disadvantaged the health of the patient.
2. No detectable medical errors of clinical significance (no errors).
3. Inconclusive.

The phrase “may have led to” reflects the fact that no objective post-encounter information was gathered, and is in agreement with the Norwegian legislation on reprimanding physicians in medicolegal cases [20]. In this legislation, a medicolegal error is defined as applicable when the physician’s action may potentially significantly harm the patient. The category inconclusive consists of medical records with content that did not make it defensible to conclude whether a medical error had occurred or not.

A medical audit was employed by using an experienced GP as a co-assessor (KS) to the first author (SZB). The two assessors discussed the inclusion of cases throughout the assessing process. In this way, the potentially controversial cases were picked out for peer review by the assessor and the co-assessor, for example, penicillin or broad-spectrum antibiotics or none, indication for hospitalization, etc.

### Variables

The following characteristics regarding the physicians were used: gender, seniority, citizenship at authorization as physician (Norwegian or non-Norwegian), specialty in GP, and seniority. The physician identities and workload were extracted from the medical records, from the fourteen-day period prior to the consultation that elicited the complaint. By this, one consultation was extracted for each physician in both groups. The other characteristics were obtained from the LSR.

The workload at the PCEU was defined as the extent of patient contacts and calculated as the number of patients divided by the number of work shifts and was grouped into five categories. The first category consisted of those having no work shift during the fourteen-day period prior to the index consultation. The remaining four categories were divided into quartiles defining workload: Low (1 to <6.6 no. of patients/no. of work shifts), Medium-low (6.6 to < 8.7), Medium-high (8.7 to < 12.0), and High (12.0 and higher).

### Statistical analyses

The data used in this paper was based on material from a previous case-control study in which the case physicians had evoked a complaint [16]. In the current study, the medical records have been assessed to study physician factors associated with medical errors. For this, we utilized the case-control data by analyzing cases (i.e. having evoked a complaint) and controls (i.e. random sample) separately.

Numbers, percentages, means, and standard deviations (SD) were provided to describe the data. Associations between assessment of errors and the characteristics of the physicians and workload were tested by Chi-Square and *t*-tests. Due to low numbers in some categories of workload, Fisher’s exact test was applied in analyses of workload. The tests were done separately for the group that evoked a complaint and for the random sample group. The data were analyzed using Statistical Package for the Social Sciences (SPSS) (Version 25). Level of significance was set to *α* = 0.05.

### Ethical considerations

The data collection was subjected to ethical considerations, and consent was obtained to retrieve personal sensitive information from the medical records (2013/99/REK vest – Regional Committee for Medical and Health Research Ethics West). This approval gave access to the medical records with the UPINs, and thereby the parameters on workload. Through this approval, the register data in the LSR were made available by the proprietor. All transmission of information was encrypted using Secure File Transfer Protocol.

The premise for the collection of person-sensitive data was that the patients should be uniformly informed in writing about the project and that the identity of the patients and physicians would not be made known to the research group. The same procedure on information had to be applied to the potentially participating physicians. The societal benefit of the project was thereby considered to justify obtaining the described information. According to these preconditions, the data material was deidentified after retrieval of the necessary data.

## Results

Table 1 shows the distribution of the three assessment categories. In the group of physicians who had evoked a complaint, 53.2% of the medical records were classified as disclosing medical errors that may have led to harm, or been disadvantageous, to the health of the patient. In the random sample group, this percentage was 3.2. The proportion of inconclusive was similar for both groups (29.9 and 27.6%). No error was the conclusion for 16.9% in the compliant group. In the random sample group, this percentage was 69.1.

**Table 1.** Assessments of medical errors in ten primary care emergency units in Norway, 2015–2017.

	Complaints	No-complaints	
	Cases	Controls	Total
Variables	*n* (%)	*n* (%)	*n* (%)
Medical errors	41 (53.2)	7 (3.2)	48 (16.3)
No-errors	13 (16.9)	150 (69.1)	163 (55.4)
Inconclusive	23 (29.9)	60 (27.6)	83 (28.2)
	77	217	294

A case-control study of complaints was used as a point of departure.

The distribution of assessments of the information in the medical records by physician characteristics and workload are presented in Table 2 for the complaint group and in Table 3 for the random sample group. In the complaint-group female physicians had a higher percentage of no-errors (30.8%), and a lower percentage of medical records assessed as inconclusive (15.4%), compared to male physicians (*p* = 0.027). However, there were no gender differences regarding medical records assessed as medical errors. No significant differences were found for the other variables (Table 2). The percentages of medical errors in the random sample group were 4.7 for female physicians and 2.3 for their male colleagues. There were no significant differences in this random sample group (Table 3).

**Table 2.** Assessments of medical errors in ten primary care emergency units in Norway, 2015–2017.

	Assessments of medical records						
	Medical errors		No-errors		Inconclusive		
Variables	*n* (%)	Mean (SD)	*n* (%)	Mean (SD)	*n* (%)	Mean (SD)	*p*-value
Gender							
Female	14 (53.8)		8 (30.8)		4 (15.4)		0.027
Male	27 (52.9)		5 (9.8)		19 (37.3)		
Seniority		8.7 (9.3)		4.3 (3.2)		8.4 (8.3)	0.242
Specialty general practice							
Yes	11 (52.4)		4 (19.0)		6 (28.6)		0.951
No	30 (53.6)		9 (16.1)		17 (30.4)		
Citizenship at authorization							
Norwegian	28 (56.0)		7 (14.0)		15 (30.0)		0.636
Non-Norwegian	13 (48.1)		3 (22.2)		8 (29.6)		
Workload^a^							0.638^b^
Only one duty	16 (57.1)		5 (17.9)		7 (25.0)		
Low	9 (69.2)		1 (7.7)		3 (23.1)		
Medium low	3 (30.0)		3 (30.0)		4 (40.0)		
Medium high	3 (33.3)		2 (22.2)		4 (44.4)		
High	10 (58.8)		2 (11.8)		5 (29.4)		

A case-control study of complaints was used as a point of departure. Group of complaints.

^a^First row the no. for just one duty in the fourteen day period. The following four rows has the quartiles of no. of patients/no. of duties: 1.0–6.6; 6.6–8.7; 8.7–12.0 and >12.0.

^b^Fischer’s exact test.

**Table 3.** Assessments of medical errors in ten primary care emergency units in Norway, 2015–2017.

	Assessments of medical records						
	Medical errors		No-errors		Inconclusive		
Variables	*n* (%)	Mean (SD)	*n* (%)	Mean (SD)	*n* (%)	Mean (SD)	*p*-value
Gender							0.472
Female	4 (4.7)		61 (70.9)		21 (24.4)		
Male	3 (2.3)		89 (67.9)		39 (29.8)		
							0.549
Seniority		6.3 (5.1)		9.1 (8.2)		11.1 (9.4)	0.192
Specialty general practice					0.065		
Yes	3 (3.3)		56 (60.9)		33 (35.9)		
No	4 (3.2)		94 (75.2)		27 (21.6)		
Citizenship at authorization							0.277
Norwegian	4 (2.4)		112 (67.9)		49 (29.7)		
Non-Norwegian	3 (5.8)		38 (73.1)		11 (21.2)		
Workload^a^							0.662^b^
Only one duty	2 (4.8)		26 (61.9)		14 (33.3)		
Low	1 (2.3)		32 (74.4)		10 (23.3)		
Medium low	1 (2.1)		36 (76.6)		10 (21.3)		
Medium high	1 (2.1)		34 (72.3)		12 (25.5)		
High	2 (5.3)		22 (57.9)		14 (36.8)		

A case-control study of complaints was used as a point of departure. Random sample group.

^a^First row the no. for just one duty in the fourteen-day period. The following four rows has the quartiles of no. of patients/no. of duties: 1.0–6.6; 6.6–8.7; 8.7–12.0 and >12.0.

^b^Fischer’s exact test.

There were 68 physicians that evoked complaints, seven physicians had two complaints and one physician had three complaints. Among the 68 physicians, 28 also contributed as control physicians. Further, 139 physicians were only in the random sample group.

## Discussion

In this study on medical errors in Norwegian PCEUs, the essential finding was related to the gender of the physicians. Female physicians in the group who had evoked a complaint were assessed to have a higher proportion of no-errors and a lower proportion of records that were inconclusive for management assessments compared to their male colleagues. Seniority, citizenship, GP specialty and workload were not significantly associated with the outcome of the assessments of the medical records. In the random sample group there were no significant differences related to the included variables.

In a previous paper on complaints, based on the same material, we found that there was no gender difference associated with the risk of evoking a complaint [16]. In that study, the medical records were not assessed regarding medical errors. Other studies have revealed a male predominance in making medical errors [3,12,13,22]. This has been correlated with nonprofessional issues, such as female physicians working fewer hours than their male colleagues and having different work and practice types [3]. Nevertheless, the gender difference has been stated as fundamental in a systematic review and meta-analysis [3]. The underlying reasons may be the perceived female characteristics of empathy, self-knowledge, and communication skills [23–26]. In the current study, we found no gender differences regarding assessments of the medical records in the random sample group. However, in the group of physicians who had evoked a complaint, the gender differences were related to no errors and inconclusive assessments. This may indicate differences in journaling between female and male physicians.

Adequate journaling is mandatory to assess the quality of medical interventions and patient care. The fact that our study revealed gender differences related to no-errors and inconclusive assessments may indicate that female physicians are more thorough in journaling than their male colleagues. These findings may also coincide with a presumed group of male physicians with generally poor clinical performance, who often elicit complaints that reveal poor journaling, making the proper clinical assessment of their performance difficult.

The medical record should contain the necessary information that is relevant to the patient’s reason for the encounter. With this, the medical record stands out as being the crucial tool for the physician to make the right decisions for the patient [27].

Physician training expressed by seniority, workload or GP specialty, did not seem to have significant implications. In a previous paper, we discussed the finding related to the absence of importance of experience expressed by seniority as a doctor [16]. The studies revealing higher rates of medical errors with increasing seniority were not confirmed by our study [28,29].

It could be anticipated that increasing experience would simplify and improve the professional decision-making process. In this context, it should be expected that having more than one work shift during a fourteen-day period, would achieve the effect of training [30,31]. However, the fourteen-day period may have been too short to reveal such an effect. On the other hand, a heavy workload did not contribute to medical errors. This may have been facilitated by better knowledge of the routines and the cooperative relations.

A conceivable reason for not confirming the advantageous effect of the GP specialty may be the overall effect of the Norwegian qualification requirements for unrestricted work in a PCEU [18]. Since 2012 a course on emergency medicine has been required for being qualified as a GP specialist [18]. This all may be of decisive importance; while 57.6% of the physicians in the random sample group had this specialty, and the additional number of physicians in training for being qualified is unknown. On this background the persistence of the gender difference is remarkable.

Language skills and cultural competence have been shown as a prerequisite for satisfactory communication, avoiding unfortunate events [12,13]. Physicians who do not have Norwegian citizenship, may have their communication skills influenced by their native language and a divergent approach on the cultural basis in communicating with patients. Nevertheless, in studying patient complaints, citizenship did not seem to be an explanatory factor or significantly associated with the risk to evoke a complaint [16]. Physicians with non-Norwegian citizenship may probably enhance their Norwegian communication skills during the years they work in Norway. However, our material did not allow any conclusions on associations between citizenship and increasing seniority.

Compatible results for physicians with or without Norwegian citizenship, may be promoted by the Norwegian prerequisites for working in a PCEU and the required course in emergency medicine for getting qualified as a GP specialist [18]. The consequence of these regulations is in line with the results of a study including graduates from foreign versus US medical schools, showing better patient outcomes with graduates from foreign schools [32]. This is explained by a rigorous approach to incorporating international medical graduates.

The physician’s attitude may induce a complaint. In this study, complaining about the behavior of the physician was not included. We acknowledge the fact that rudeness may be experienced as harmful. This is an important issue in ordinary general practice, where building trust and confidence are crucial parameters for following up. However, we doubt that this kind of behavior may be significantly medically harmful to the patient. We recognize that poor communication can cause the patient to omit symptoms or the doctor may omit follow-up questions. To unravel if these unfortunate conditions have been present medical records from follow-up consultations must be available. This was, however, not within the scope of the given ethical considerations.

It is intriguing that for only 53.2% of the patient complaints a medical error was uncovered. This is consistent with a Norwegian study on medicolegal assessments [15]. However, this does not support the assumption that nearly half of the complaints were unfounded. In the same way, disclosing sparse recording does not necessarily lead to the conclusion that the medical measures have been erroneous. As medical records in PCEUs often do not document the complete course of events, this inconclusiveness may be hiding deficiencies in managing the patients. These deficiencies may be assumed as the main reason for the proportion of medical records assessed as inconclusive in this study, i.e. making it inadequate to decide whether or not a medical error could have or had led to patient harm.

Furthermore, the quality of medical records is measured by the covering of relevant and necessary information [21]. The finding that only 3% of the medical records in the random sample group revealed medical errors, is consistent with larger studies from primary health care [1,10].

As complaints and errors should be seen in relation to each other, the lack of concurrence in the results of our studies may be surprising [16]. However, as a complaint is written in retrospect, the medical record written in connection with the consultation should be the basis for the assessments.

Recently, a Norwegian study of the frequency and distribution of disciplinary actions for medical doctors found higher rates for physicians who work in small clinics or alone (GPs and private specialists) than for those working in large organizations (hospital doctors) [33]. This emphasizes the impact of systemic and structural factors. However, the study design does not allow any conclusion related to the differences of impact of organizational or systemic factors on the decisions of disciplinary actions. The reported differences may thus partly be attributed to how these factors affect the assessment of the cases, for GPs and hospital doctors.

In the current study, assessing journaling has presented itself as a cornerstone in learning from medical errors. Through further studies, the elements of the medical record should be analyzed to uncover the critical elements in obtaining information about the patient's reason for encounter and implemented measures. This means studying the underlying elements in the physician’s considerations and decisions documented in the medical record, including any additional notes revealing information that may have been available to the physician. This should include testing medical history–taking devices with the potential to increase the quality of anamnesis and differential diagnosis [27].

To learn, we must use current knowledge and do more research to gain more knowledge to identify why errors occur. This must include the impact of factors like communication skills, behavior, and decision-making ability. Out-of-hours services need to focus on culture for learning, acknowledging the need for basic routines, and good leadership guided by qualitative studies. This may unravel the impact of factors like communication skills and behavior.

The design and results of this study on medical errors may have the potential of guiding further research and facilitate reflection on drivers for improvements.

### Strengths and limitations

The strength of this study is that it includes a group of physicians evoking a complaint and a random sample group, both groups with valid and nearly complete data sets. The proportion of inconclusive medical records was similar in the complaint group and the random sample group (29.9 and 27.6%). This substantiates the assumption of consistency in the assessments of the medical records in the two groups. Knowing about the complaints does not seem to have influenced the judgements. The use of a normative tool facilitated consistency in reviewing the medical records [20].

The main weakness of the study is the unexpected low number of medical records included. There were several reasons for this: compatibility problems with the customized data extraction programme and the different electronic medical record systems, change of leadership during the study period at some PCEUs and heavy workload. The lack of electronic compatibility was the essential reason for one of the larger units. Broad-scale extraction of textual material from different electronic medical record systems is at present still not possible.

The low number of medical records creates limitations for the application of the results of this study. It is a weakness of the study that we were not able to study communication problems and cultural competence among the participants.

As smaller PCEUs with rather few participating physicians were included, the frequency of work shifts increases the probability to be picked up as control more than once. This may be a bias in this study, reflected by the lower number of individual physicians than should be expected from the number of cases. This does, however, not seem to have influenced the results.

This study includes both a group of physicians evoking a complaint and a proper random sample group. It is a strength that both groups had valid and nearly complete data sets. However, we acknowledge the limitations of this material, as cases and controls were analyzed separately.

## Conclusions

In studying physician factors that may induce medical errors in PCEUs in Norway, medical records written by two groups of physicians were reviewed: a group of physicians who had evoked a complaint and a random sample of physicians. The only significant results were found in the complaint group. In this group, we found a higher percentage with no assessed medical error and a lower percentage with assessments of inconclusive medical records among female physicians compared to their male colleagues. Physician gender, seniority, citizenship, GP specialty, or workload were not significantly associated with assessed medical errors in a random sample of physicians. The Norwegian regulations on working in a PCEU, may have modulated the results. Future research should focus on the underlying elements of these findings, including journaling, organizational and structural factors.

## Data Availability

The permission was granted provided de-identification or eradicating the study material within the end of 2018. De-identification of the study material was performed in due time. The datasets used during the current study are available de-identified from the corresponding author on reasonable request.
